# Outcomes of Surgical Repair of Tetralogy of Fallot: A Comparison Between the Adult and Pediatric Population

**DOI:** 10.7759/cureus.41467

**Published:** 2023-07-06

**Authors:** Meenal S Khan, Azam Jan, Haseeb Ahmed, Mudassar Khan, Ahmad D Khan, Rafat Shakil, Bahauddin Khan, Zarkesha Aman, Waleed S Ali, Ahmad Mahmood

**Affiliations:** 1 Surgery, Rehman Medical Institute, Peshawar, PAK; 2 Cardiothoracic Surgery, Rehman Medical Institute, Peshawar, PAK; 3 Cardiothoracic and Vascular Surgery, Rehman Medical Institute, Peshawar, PAK; 4 Emergency Medicine, ER of Dallas, Dallas, USA; 5 Endodontics, Sardar Begum Dental College, Peshawar, PAK; 6 Ophthalmology, Hayatabad Medical Complex, Peshawar, PAK

**Keywords:** tetralogy of fallot, patent ductus arteriosus, mortality, cardiopulmonary bypass, intensive care unit, congenital cardiac surgery, pulmonary valve, cardioplegia, right ventricular outflow tract, ventricular septal defect (vsd)

## Abstract

Introduction

Tetralogy of Fallot (TOF) is the most common cyanotic congenital heart disease. Early detection and timely treatment have provided successful repair of the anomaly in the developed world. However, in the developing world, there is still a burden of uncorrected TOF patients reaching adulthood. The goal of this study is to determine whether there is any difference in postoperative complications between adult and pediatric populations following surgical correction for TOF.

Methods

This study involved all those patients who received primary or secondary surgical repair for TOF in our facility between January 2017 and December 2020. The patients were split according to their age into the pediatric group if they were under 18 years and the adult group if they were 18 years or older. Patients with absent pulmonary valve or pulmonary atresia were not included in this study. Patients with large major aortopulmonary collateral arteries (MAPCA) were also excluded from this study. All patients underwent total correction through a median sternotomy approach. The ventricular septal defect was closed with a Bard knitted fiber patch. The right ventricular outflow tract (RVOT) was augmented by excising muscle bands or fibrous bands in the RVOT. If the annulus was smaller than the 3.5 z score, then a transannular patch was done using an autologous pericardium. The main pulmonary artery was augmented in every surgery using an autologous pericardial patch. All patients were shifted to the ICU on the ventilator and were extubated after fulfillment of the extubation criteria. Postoperative complications measured included re-opening, re-intubation, prolonged ventilation (>24 hours), and mortality within the index hospital admission. The clinical data of all patients were prospectively collected and analyzed using the chi-square test and t-test. A p-value of less than or equal to 0.05 was considered significant.

Results

The total number of patients was 134. This included 83 males (60.1%). A total of 114 patients who were aged below 18 years were included in the pediatric group, and 20 patients aged equal to or more than 18 years were included in the adult group. The mean average perfusion time in minutes in the adult group was 125.8 and in the pediatric group, it was 98.79. Similarly, the mean average of the cross-clamp time was also longer in the adult group at 89.55 minutes versus 69.63 minutes in the pediatric group. Overall, in the adult group, three (15%) patients had postoperative complications, while in the pediatric group, a total of 14 (11.9%) patients had postoperative complications (p = 0.001). However, there was no significant difference in the number of re-openings (8.5% vs. 10%; p = 0.8). The total mortality observed was 16 (11.59%). This included 14 (11.9%) in the pediatric group and two (10%) in the adult group. There was no significant difference between the two groups (p = 0.8).

Conclusions

Surgical repair of TOF can be performed in both adult and pediatric populations with acceptable outcomes. The mortality rate was found to be slightly greater in the pediatric population compared to the adults. However, it can be seen that the number of postoperative complications is greater in adults. Further research is needed to optimize outcomes for both pediatric and adult patients with TOF.

## Introduction

Tetralogy of Fallot (TOF) is one of the first cyanotic congenital cardiac diseases to be successfully corrected by cardiac surgeons [1]. It makes up to 7-10% of all cardiac diseases that are present by birth and have a significant fatality rate if left untreated. The patients who are left untreated have a survival rate of only 24% [2].

A combination of four congenital cardiac defects leads to TOF. The four anatomic features that make up the TOF are hypertrophy of the right ventricular, obstruction of the right ventricular outflow tract (RVOT), ventricular septal defect (VSD), and overriding of the aorta with an occurrence of 3.9/10,000 births [3]. The severity of RVOT obstruction characterizes the post-natal presentation of the disease, with the severity of the obstruction directly proportional to the severity of cyanosis [4].

There are three main ways to go about the surgical treatment of TOF. The first approach is palliative in the form of the assembly of a systemic to pulmonary artery shunt, which is then followed by definite repair later. The other methods are either total correction surgery or primary open-heart surgery [5]. The operative mortality of TOF is decreasing dramatically [6]. Untreated TOF is becoming a rarity in developed countries due to its early diagnosis and treatment in infancy; however, unrepaired TOF patients have a short natural history, with 25% dying by the age of one, 40% by the age of three, and a massive 70% by the age of 10 years [7]. The percentage of early mortality among adults and pediatrics was 7.55%, which is almost the same in both age groups in a study done at Erasmus University Medical Center [8], while a study done by Erdoğan et al. had a mortality of only 3.1% [9]. A study conducted by Hashemzadeh et al. had a slightly higher in-hospital mortality rate of 6.9% [10].

The purpose of our study was to analyze the outcome of TOF surgery in the pediatric and adult population.

## Materials and methods

A retrospective cross-sectional study was carried out at a tertiary care hospital following patient permission and approval from our hospital's ethical review committee (Rehman Medical Institute, Research Ethics Committee; Ref. No. RMI/RMI-REC/Article Approval/76, September 07, 2022). This study includes all the patients with corrective surgery for TOF from the year 2017 to 2020 in the cardiothoracic section of a hospital providing tertiary care. We further sub-classified our patients into adults and pediatrics. Pediatrics included patients whose age was below 18 years while adults included those who were aged 18 years and above. Different preoperative, intraoperative, and postoperative parameters were compared among them, which included any associated disorders the patients had along with TOF such as atrial septal defect (ASD) and patent ductus arteriosus (PDA), or any previous palliative repair such as Blalock-Taussig shunt or Glenn shunt. We also compared the outcome of different types of patches being used in the surgery and looked into the early outcome of the surgery, that is outcomes during the hospital stay of the patient. Data were collected from the cardiothoracic surgery ward archives, which were gathered on data collection form and then exported in Microsoft Excel (Microsoft Corporation, Redmond, WA) format, which was then converted into SPSS (IBM Corp., Armonk, NY) format. All the results were then represented in the form of tables, and the t-test and chi-square test were used, keeping the p-value < 0.05 as significant. Currently, at our institute, we have three treatment options for TOF patients depending on their anatomy, age at presentation, and symptoms, and surgical planning is done accordingly: (1) neonates/infants with no cyanosis: wait till one year and then offer surgery for correction; (2) neonates/infants with cyanosis and unstable: offer palliative procedure and consider for surgical correction after infancy; (3) primary corrective surgery as the first option.

Our current practice includes options 1 and 2 only, as we have a minimum age for corrective surgery at our hospital of three years. So all the pediatric patients included in this study were aged more than three years.

## Results

A total of 134 patients had corrective surgery for TOF ranging from the year 2017 up to 2020 in the cardiothoracic department of a tertiary care hospital.

Table 1 shows that among these patients, 83 (60.1%) were male and 41 (39.9%) were female. The average age of the pediatric patients (in years) was fairly young (mean = 9.20, SD = 4.478). The same was the case for adult patients (mean = 25.65, SD = 8.77). The height (in centimeters) of the pediatric population was mean = 122.59 and SD = 33.74, as well as the height of adults was mean = 156.5 and SD = 13.2. The weight (in kilograms) of both the pediatric (mean = 25.166, SD = 12.95) and the adult population (mean = 51.85, SD = 13.3) has also been mentioned in Table 1.

**Table 1:** Preoperative parameters for TOF corrective surgery patients TOF: tetralogy of Fallot; std: standard deviation.

Serial No.	Variables	Pediatrics (age < 18)	Adults (age =/> 18)	P-value
1	Total TOF patients	114	20	
2	Age (mean +/- std)	9.20 +/- 4.478	25.65 +/- 8.77	0.0
3	Weight in kg (mean +/- std)	25.166 +/- 12.95	51.85 +/- 10.46	0.0
4	Height in cm (mean +/- std)	122.59 +/- 33.74	156.50 +/- 13.20	0.0
5	Male	71 (60.2%)	12 (60%)	

Table 2 shows that the mean perfusion time and cross-clamp time were higher in adult patients as compared to pediatric patients. All the pediatric and adult patients received intraoperative blood products, which included red blood cells, fresh frozen plasma, and platelet units. Intraoperatively, we looked at the type of patch used, which concluded that 13% of the pediatric patients had no patch against 15% of adult patients, and adult patients more commonly had transannular patch while pediatric patients had got main pulmonary artery (MPA) patch. In our study with TOF, we noted additional associated cardiac disorders, including PDA and ASD. Among the TOF patients, four pediatric cases were identified with concurrent PDA, while only one adult case presented with PDA. Furthermore, two pediatric TOF patients were found to have associated ASD, while only one adult TOF patient had ASD. Regarding previous palliative procedures, a retrospective analysis revealed that a total of eight TOF patients had undergone a Blalock-Taussig (BT) shunt repair in the past. Of these, seven cases were in the pediatric group, whereas only two cases were observed in the adult population. Please note that this information is based on our specific patient population and may not be generalizable to other cohorts.

**Table 2:** Intraoperative parameters for TOF corrective surgery patients TOF: tetralogy of Fallot; Std.: standard deviation; PRBC: packed red blood cells; FFP: fresh frozen plasma; RVOT: right ventricular outflow tract; PA: pulmonary artery; MPA: main pulmonary artery; PDA: patent ductus arteriosus; ASD: atrial septal defect; BT shunt: Blalock-Taussig shunt.

Serial No.	Variables	Pediatrics (age < 18) (N = 114)	Adults (age =/> 18) (N = 20)	P-value
1	Perfusion time in minutes (mean +/- std.)	98.79 +/- 32.77	125.80 +/- 56.22	0.003
2	Cross-clamp time in minutes (mean +/- std.)	69.88 +/- 26.66	89.55 +/- 45.09	0.008
3	No. of patients receiving intraoperative blood products	114 (100%)	20 (100%)	0.75
4	No. of patients receiving PRBC	108 (94.7%)	17 (85%)	0.176
5	No. of patients receiving FFP	109 (95.6)	17 (85%)	0.001
6	No. of patients receiving platelets	109 (95.6)	17 (85%)	<0.001
7	No RVOT/PA patch	15 (13.3%)	3 (15%)	
8	Transannular patch	44 (38.5%)	10 (50%)	
9	MPA patch	55 (48.2%)	7 (35%)	
10	Associated PDA	4 (3.6%)	1 (5%)	
11	Associated ASD	2 (0.91%)	1 (5%)	
12	Previous BT shunt	7 (6.1%)	2 (10%)	

Table 3 shows the postoperative parameters where the total number of patients receiving blood products in the pediatric age group was 60 while that used in adults was eight. We looked at the postoperative complications that included reopening for bleeding, postoperative stroke, pleural effusion, heart failure, multi-system failure, atrial fibrillation, ventricular tachycardia, reintubation, prolonged ventilation for more than 24 hours, and mortality that showed that the percentage of pediatric patients having postoperative complications was 11.9% while 15% of adult patients had postoperative complications present. We have a slightly higher mortality rate in our pediatric group (11.9%) as compared to adults (10%). This includes the in-patient mortality that was observed in index admission before discharging the patient from the hospital.

**Table 3:** Postoperative parameters for TOF corrective surgery patients

Serial number	Variables	Pediatrics (age < 18) (N = 114)	Adults (age =/> 18) (N = 20)	P-value
1	Number of patients receiving blood products postoperatively	60 (52.6)	8 (40)	0.297
2	Number of patients receiving red blood cells	40 (35)	7 (35)	0.289
3	Number of patients receiving fresh frozen plasma	37 (32.4)	5 (25)	0.276
4	Number of patients receiving platelets	25 (21.9)	4 (20)	0.223
5	Number of patients having postoperative complications	14 (9.6)	3 (15%)	0.736
7	Reintubated	2 (1.7%)	0	0.551
8	Reopening for bleeding	10 (8.5%)	2 (10%)	0.859
9	Prolong ventilation > 24 hours	2 (1.7%)	0%	0.551
10	In-patient mortality	14 (11.9%)	2 (10%)	0.772

## Discussion

Since its introduction in the 1950s, the result of comprehensive correction of TOF has improved dramatically [11], One-stage total repair surgery is the ideal surgical treatment used to treat symptomatic TOF, ensuring that sinus rhythm is maintained, that there is no longer any obstruction in the RVOT, that the pulmonary valve of the patient is still functional, additionally, that the VSD has been totally repaired [12]. Children with TOF are operated at the age of one year, with most repairs done before the age of six months, voluntarily. If the blockage to the RVOT is not significant, typically, the intracardiac repair is postponed until beyond the neonatal period, to give time for resistance of the pulmonary vessels to decrease and the child to put on weight [13].

With enhanced intraoperative and postoperative care, Kirklin performed the first successful pump oxygenator repair at the Mayo Clinic. Surgical risk for many congenital anomalies has decreased, and it is becoming obvious that early correction vs. palliation has multiple advantages. Two-stage surgery patients have a cumulative death rate of 28.4% (27/95), which is significantly greater than primary repair patients (11.1%; p = 0.001) [14]. Only seven patients from the pediatric group while two adults in our research had formerly undergone a palliative procedure, and the remaining underwent a primary repair when they grew older. Among many advantages of early repair, as shown by studies and its direct effect on the myocardium, studies also showed that it protects the brain from chronic hypoxia [15]. Hence, early surgical correction is recommended in TOF patients.

When the narrowed pulmonary annulus is inadequate to warrant a complete correction, transannular patch (TAP) repair is one of the most efficient ways to widen the ROVT [16]. In our study, the TAP was used in 38.5% of neonatal cases and 50% of adult TOF cases, which was comparatively very high than a study done in Faisalabad where the TAP was used in only 22% of patients but similar to a study done by Athanasiadis et al. where the TAP was used in 64.7% patients [17]. In our study, MPA patch plasty was done in most of the neonatal TOF cases, the percentage being 48%, and only 35% of adult patients got MPA patch plasty done.

The surgical mortality in our study was higher (11.9%) in the pediatric group as compared to 10% in the adults’ group, which was almost similar to the mortality rate (8.8%) of a study done in Afghanistan [18] and lower than the surgical mortality in another study done in Brazil, which was found to be 15% [19].

In our pediatric group, the average time for a cardiopulmonary bypass is 98.79 (32.77) minutes while in adults it is 125.80 (56.82) minutes. The aortic cross-clamp time recorded in our study was 69.88 (26.66) minutes for pediatric patients and 89.55 (45.09) minutes for adults, respectively, which, as compared to a study done in Karachi, is comparatively low [20], but higher as compared to a study done by Kolcz et al. at the Alfred I. DuPont Hospital [21].

Patients with a corrected TOF, on the other hand, may experience difficulties later, including heart failure, arrhythmias, severe pulmonary regurgitation, and sudden cardiac death, while the long-term effects of early repair are still unknown [22]. To reduce the incidence of late acute severe pulmonary regurgitation, many of our patients receiving MPA patch also got pulmonary valve reconstruction using autologous pericardial patch depending on surgeons’ preference.

In developed countries, primary surgical correction is usually done between the age of three to 12 months but due to a variety of factors, including delay in diagnosis, financial limitations, higher morbidity, and mortality due to lack of medical skills in underdeveloped countries, this is not a usual practice. Hence, accurate data on the morbidity and mortality of surgically corrected TOF cases are currently scarce in our countries [23].

The realization of "safe elective repair in early infancy" of TOF is evidence in favor of early surgical correction and its outcomes with acceptable mortality, according to Di Donato et al. [24]. Determining and reducing the necessity of repeat surgery requires more effort. Mortality and morbidity long-term surveillance, with an emphasis on the function of the right ventricle and arrhythmias, is needed to explain the advantages of prompt repair in the long run.

## Conclusions

Surgical repair of TOF can be performed in both adult and pediatric populations with acceptable outcomes. The mortality rate was found to be slightly greater in the pediatric population compared to the adults. However, it can be seen that the number of postoperative complications is greater in adults. Further research is needed to optimize outcomes for both pediatric and adult patients with TOF.
